# Interruption of the intestinal immune route to the systemic circulation associated with early hyperhydration minimized splanchnic microcirculation damage and improved sepsis survival

**DOI:** 10.1186/cc12971

**Published:** 2013-11-05

**Authors:** Fernando M Dulcini, Ana MA Liberatore, Bianca C Zychar, Ivan HJ Koh

**Affiliations:** 1 Department of Surgery, Federal University of São Paulo, Brazil; 2 Department of Pediatrics, Federal University of São Paulo, Brazil

## Background

Considering that the communication of the intestinal immunity with the systemic bloodstream can be a relevant adjuvant factor in the amplification of the host systemic inflammatory response and subsequent multiple organ dysfunction in sepsis, we aimed to evaluate the effect of the obstruction of the mesenteric lymph duct (OMLD) associated with massive fluid therapy in the early phase of sepsis and severe sepsis models.

## Materials and methods

Adult Wistar-EPM rats were submitted to 10^8 ^(S8) or 10^9 ^(S9) CFU/ml *Escherichia coli *inoculum intravenously (i.v.) (DL_80 _within 26 hours), and were treated with hyperhydration (HH) with or without previous OMLD (*n *= 5/group). Control group were naïve animals (N) and animals submitted to HH or sepsis only. The mortality of groups was followed up to 30 days after experiments and microcirculation monitoring was observed at 6 hours post sepsis induction by videomicroscopy (sidestream darkfield imaging (SDF)).

## Results

**Figure 1:**
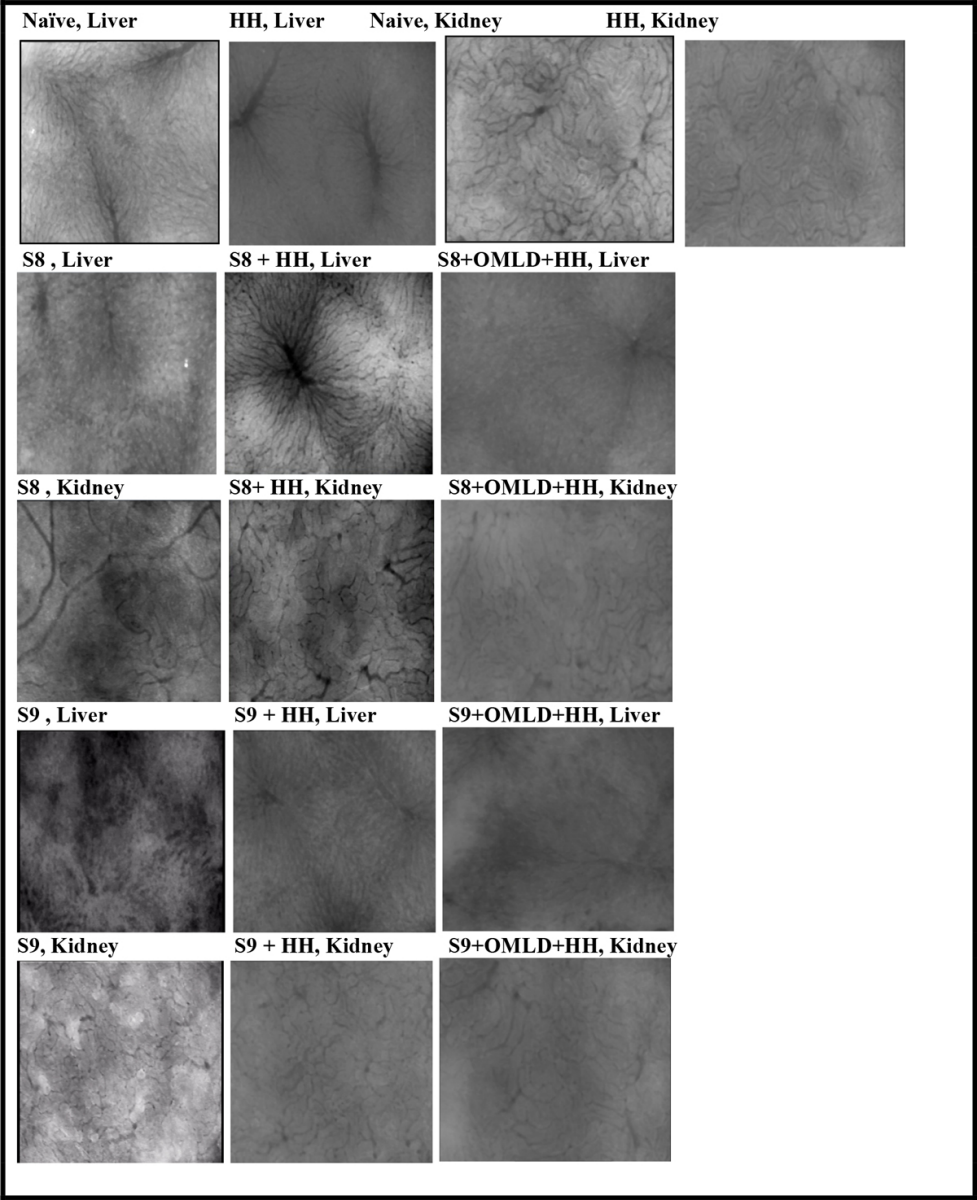
**Splanchnic organ's microcirculation following 6 hours after sepsis or HH procedures by SDF monitoring: (a) HH; (b) S8 + OMLD + HH; (c) S9 + OMLD + HH**.

The effect of OMLD + HH reduced significantly the sepsis mortality rate: S8 (60% to 14.5%) and S9 (80% to 60%). Besides, the liver and kidney microcirculatory features were better preserved as compared with untreated sepsis groups under video-microscopy (SDF) monitoring. (Figure 1).

## Conclusions

These preliminary findings showed that both HH and OMLD have a potential therapeutic application in sepsis by minimizing the splanchnic organ's microcirculation dysfunction.

